# A novel mutation in 3’UTR of *GJB2* gene in autosomal recessive nonsyndromic sensorineural hearing loss in South Indian population

**DOI:** 10.1186/1755-8166-7-S1-P113

**Published:** 2014-01-21

**Authors:** Maria sebastian, Praveena Davis, Padmaja Ramdas, Moinak Banerjee

**Affiliations:** 1 Human Molecular Genetics Laboratory, Rajiv Gandhi Centre for Biotechnology, Kerala, India; 2 National Institute of Speech & Hearing, Kerala, India

## Background

Autosomal recessive nonsyndromic sensorineyral hearingloss also known as “DFNB” causes 20% of all childhood deafness and may have carrier rate as high as 2.8%. Fifty to eighty percent of DFNB cases causing severe to profound hearing impairment, results from mutations in a single gene, GJB2(DFNB1), that encodes the protein connexin 26(Cx26) located on chromosome 13q11-12. Aim of this study was to explore the status of reported pathogenic mutation as well as novel variants in South Indian population.

## Material and methods

Promoter region, exons and 3’UTR of GJB2 gene were screened to find mutations associated with autosomal recessive nonsyndromic deafness from 50 families speaking Dravidian Malayalam language. Primers were designed using primer Z software for sequencing promoter, exons as well as 3’UTR of GJB2 gene. Mutation surveyor software was used to find changes in the sequence.

## Results

Pathogenic mutations in the coding exon like W24X, R127H, M163V were found in our study population. W24X mutation was the most commonly found mutation causing Stop codon. Screening of 3’UTR region of *GJB2* lead us to find a novel mutation in this region located 1031 bases downstream of the gene causing a change from G to A. Bioinformatics analysis using different miRNA prediction tool like Microsniper, mirSNP suggest, this change causing a differential binding of miRna including hsa-miR-924, hsa-mir-501-5p,hsa-mir-1225-3p,hsa-mir-558 and hsa-mir-615-3p. Further it was revealed that this mutation indeed causes changes in expression of this gene.

## Conclusions

The present study could find a novel mutation in the 3’UTR region 1031bp downstream in GJB2. Bioinformatic analysis provides evidence for functional implication of this mutation which might have a role in pathogenicity of the disease. This study could also validate the importance of reported mutations in our population with W24X to be the common pathogenic mutation found in this population.

